# A Review of Its Poor-Law Medical Work

**Published:** 1915-04-24

**Authors:** 


### THE REPORT OF THE LOCAL GOVERNMENT BOARD.

### A Review of its Poor-Law Medical Work.

The most interesting part of the recently issued report of the Local Government Board for 1913-14 deals with the striking reduction in the numbers of the casual poor in London during the years 1913 and 1914. A reduction occurred in all the English and Welsh counties, but was most marked in London, as the following table shows:—

*Casual Poor.*

| | 1912 | 1913 | 1914 |
|---|---|---|---|
| London ... ... ... ... ... | 938 | 534 | 292 |
| Rest of England and Wales ... | 8,794 | 8.348 | 7,276 |

The large and continuous decrease noticeable in the case of London is attributed to the transference of the casual wards to the Metropolitan Asylums Board, and the formation of the Houseless Poor Committee, consisting of representatives of official bodies and charitable agencies This reduction has permitted the closure of some of the Metropolitan casual wards, so that now only twelve are open. In addition, the "sleeping-out" class has been almost entirely cleared from the Embankment, the numbers in the "sit up" shelters and free lodging-houses have decreased, and there has been a reduction in the casuals in the twelve Unions bordering on London and in the number of inmates in the licensed common lodging-houses.

The work of the Metropolitan Asylums Board in this connection which has been most productive of good has been the scheme of co-operation it has inaugurated with voluntary philanthropic agencies. During the year 844 persons were passed on to these agencies, in most cases on their application for admission to the casual wards, in the remainder after a night's stay. Outside London the decrease appears to be due to the introduction in many counties of the way-ticket system and the formation of Committees of Guardians, which have secured uniformity of treatment in the casual wards within the counties. Under the way-ticket system the genuine wayfarer is detained one night only, is discharged early, and given a way-ticket and a meal ticket securing him a midday meal—usually of bread and cheese—at a suitable distance on his route, and a night's lodging at a prescribed workhouse or casual ward. Similar tickets are issued to him from day to day along the route he wishes to pursue for the purpose of seeking work. The general opinion of the inspectors is that this system has reduced the amount of begging. On the other hand, Mr. Duff, the inspector for Salop, Chester, and adjoining counties, fears that the more generous diet, the midday meal, and the absence of task may be attracting to the casual wards a better class of workmen. He says: "To a man out of work and hesitating whether to take to the road, the removal of the usual deterrents to tramping may well weigh down the scales in the direction of a free pedestrian tour through the country."

A decrease is also shown as regards all other classes of paupers except the insane. The total number of persons in receipt of relief on January 1, 1914, excluding casuals and the insane, was 628,322, a decrease of 33,342 on the corresponding figures for 1913. Attention is drawn to the fact that pauperism as a whole is higher in London than in the rest of the country, but that the excess occurs entirely in the class of paupers relieved in institutions. An important factor in the causation of this is held to be the great development in London of Poor-Law infirmaries and other specialised institutions. The following table is given showing the striking decrease in the number of paupers (excluding the insane) over seventy years of age since the Old Age Pensions Act came into force:—

*Paupers over 70 relieved on January 1.*

| | Indoor | Outdoor | Total |
|---|---|---|---|
| 1910 ... ... ... | 57.701 | 138.223 | 195.924 |
| 1911 ... ... ... | 55.262 | 93.177 | 148 439 |
| 1912 ... ... ... | 49.373 | 9.530 | 58 903 |
| 1913 ... ... ... | 49,207 | 9,563 | 57,770 |
| 1914 ... ... ... | 48,103 | 8,945 | 57,048 |

### A New Return.

For the first time in these annual reports a return is given distinguishing between the number of sick in infirmaries separately administered and the number in workhouses and infirmaries still under the control of the workhouse master. The numbers are very striking, and show how much has yet to be done in the improvement of the conditions under which the sick are treated in Poor-Law institutions. In London on January 1, 1914, 14,612 patients were in separate infirmaries and 7,622 in workhouses; in the rest of England and Wales only 13,037 were in separate infirmaries, and 59,263 were still in the workhouse.

A table is given showing the amount of vacant accommodation in the Metropolitan institutions on January 1, 1914. From this it appears there were 9,604 empty beds in the workhouses, and 1,664 in the separate infirmaries. Commenting upon this, Mr. Oxley, the Inspector for the Metropolis, says: "It would seem to be desirable that the possibility of reorganisation with a view to making the most of existing accommodation should be carefully considered before fresh building is undertaken to provide additional institutions for any special class." In this connection it may be noted that the formation of the City of Westminster Union by the amalgamation of the old Unions of St. George's (Hanover Square), Strand, and Westminster enabled the workhouses in Poland Street and Sheffield Street, the schools and workhouse at Edmonton, and the children's receiving home in Milman Street to be closed, and has resulted in a saving of something like £25,000 a year after making provision for compensating the officers who have been displaced.

### The Only Medical Statistics.

As regards medical relief the report says that "during recent years there has been a marked extension of the provision of separate accommodation for the care and treatment of the sick in Poor-Law institutions, and in some districts it may perhaps be said that these institutions almost occupy the place of general public hospitals." Statistics are given of the work done in the maternity wards. 2,228 mothers were admitted, and of these seventeen died. In five cases the death was attributable to causes unconnected with childbirth, in nine to eclampsia, in one to pulmonary embolism, in one to prolonged labour before admission, and in one to sepsis. One hundred and ninety of the children were still-born, 186 of the live births were born prematurely, and there were twenty-seven cases of twins. Eighty of the infants died in the first fourteen days of life; forty-one of the deaths were due to prematurity, sixteen to congenital defects, seven to syphilis, three to difficult labour, and three to other causes.

These maternity statistics are the only medical statistics given, and no details of the medical work in the infirmaries are contained in the report. This is a serious defect. The Poor-Law medical work of the Local Government Board is one of the most important of its activities. It has at its disposal the means to present statistics in the incidence of disease which would be of the greatest value. The neglect to do so is the more striking when one compares it with the elaborate medical statistics published by the Public Health Committee of the London County Council and the Statistical Committee of the Metropolitan Asylums Board. Apart from the National Insurance Commissioners there is no other public body dealing with anything like so large a number of the sick, and the failure to use the opportunity for its statistical presentation is a serious loss from a medical point of view.

